# Uncovering the Genetic Basis of Grain Protein Content and Wet Gluten Content in Common Wheat (*Triticum aestivum* L.)

**DOI:** 10.3390/plants15020307

**Published:** 2026-01-20

**Authors:** Quanhao Song, Wenwen Cui, Zhanning Gao, Jiajing Song, Shuaishuai Wang, Hongzhen Ma, Liang Chen, Kaijie Xu, Yan Jin

**Affiliations:** 1 Zhumadian Academy of Agricultural Sciences, Henan Provincial Engineering Research Center for Germplasm Improvement and Breeding of Multi-Resistant and High-Efficiency Wheat, Zhumadian 463000, China; songmanl.2005@163.com (Q.S.); wheatls2017@163.com (Z.G.); bb20251104zmd@163.com (J.S.); sy200604@126.com (S.W.); wheatgrain1707@163.com (H.M.); 2 Zhumadian Academy of Industry Innovation and Development, Huanghuai University, Zhumadian 463000, China; cuiwen1219@nwafu.edu.cn; 3 State Key Laboratory for Crop Stress Resistance and High-Efficiency Production, Collage of Agronomy, Northwest A&F University, Yangling 712100, China; chenliang9117@nwafu.edu.cn; 4 Institute of Cotton Research, Chinese Academy of Agricultural Sciences, Anyang 455100, China

**Keywords:** common wheat, GWAS, processing quality, KASP, SNP

## Abstract

Improving wheat processing quality is a crucial objective in modern wheat breeding. Among various quality parameters, grain protein content (GPC) and wet gluten content (WGC) significantly influence the end-use quality of flour. These traits are controlled by multiple minor effect genes and highly influenced by environmental factors. Identifying stable and major-effect genetic loci and developing breeder-friendly molecular markers are of great significance for breeding high-quality wheat varieties. In this study, we evaluated the GPC and WGC of 310 diverse wheat varieties, mainly from China and Europe, across four environments. Genotyping was performed using the wheat 100K SNP chip, and genome-wide association analysis (GWAS) was employed to identify stable loci with substantial effects. In total, four loci for GPC were identified on chromosomes 1A, 3A, 3B, and 4B, with explained phenotypic variation (PVE) ranging from 6.0 to 8.4%. In addition, three loci for WGC were identified on chromosomes 4B, 5A, and 5D, which explained 7.0–10.0% of the PVE. Among these, three loci coincided with known genes or quantitative trait loci (QTL), whereas *QGPC.zaas-3AL*, *QGPC.zaas-4BL*, *QWGC.zaas-4BL*, and *QWGC.zaas-5A* were potentially novel. Seven candidate genes were involved in various biological pathways, including growth, development, and signal transduction. Furthermore, five kompetitive allele specific PCR (KASP) markers were developed and validated in a natural population. The newly identified loci and validated KASP markers can be utilized for quality improvement. This research provides valuable germplasm, novel loci, and validated markers for high-quality wheat breeding.

## 1. Introduction

Common wheat (*Triticum aestivum* L.) is one of the most important food crops globally, providing approximately 20% of the calories and 25% of the protein to humans (FAO, http://www.fao.org/faostat/en/, accessed on 12 June 2025) [1]. Grain quality improvement is a key target in wheat breeding programs [2,3]. As a major protein source, the demand for high-quality wheat is expected to rise sharply in the near future. Thus, enhancing the quality potential of wheat remains a main breeding objective [4,5,6]. Wheat quality encompasses morphological, nutritional, and processing quality [7,8]. Protein is the main component of wheat kernels, and its content and quality are key factors determining processing and nutritional quality [9,10,11]. Protein quality traits mainly include grain protein content (GPC) and wet gluten content (WGC) [12,13,14,15], which are core parameters for evaluating the intrinsic quality and processing performance of wheat, directly affecting the end use (e.g., bread and noodles) and market value of flour [16]. Although the gluten aggregation and phenolic composition are important for specific applications, GPC and WGC represent broader, heritable traits with significant economic impact. Uncovering the genetic basis of GPC and WGC is crucial for the genetic improvement of wheat quality, helping breeders create new varieties with high yield and excellent processing quality [17,18,19], and meeting the specific needs of different regions and markets [20,21,22].

Extensive genetic studies have employed diverse strategies to dissect the genetic architecture of wheat quality traits. Wheat protein quality traits are quantitative traits controlled by multiple minor genes [23,24,25]. Previous studies reported that the quantitative trait loci (QTL) controlling GPC are mainly distributed on chromosomes 2A, 2B, 2D, 3D, 5A, 6A, 6B, and 7B [26,27,28,29,30], whereas the QTL controlling WGC are mainly located on chromosomes 1A, 1B, 2A, 3A, 5A, 5B, 6B, 7B, and 7D [8,10,30,31,32]. GWAS in different populations, such as Russian spring wheat and diverse winter wheat panels, have identified multiple significant loci for GPC and WGC [31,32]. Furthermore, linkage mapping in biparental populations has pinpointed major QTLs on chromosome 1D controlling gluten aggregation properties [10]. Notably, the functional validation of specific alleles, such as NAM-A1d being linked to lower GPC, has enhanced the mechanistic understanding [31]. Subsequent meta-analyses have effectively consolidated numerous reported QTLs into refined meta-QTLs, providing more reliable genomic regions for these complex traits [30]. Despite these advances, novel loci continue to be discovered, as evidenced by the identification of a stable, high-impact QTN for gluten content on chromosome 2A derived from T. timopheevii [8], and the reporting of several novel QTLs in regional wheat lines [33], underscoring the ongoing potential for genetic discovery in this field.

However, most previous results were primarily based on a limited number of simple sequence repeat (SSR) or diversity array technology (DArT) markers, which make gene cloning and marker-assisted selection (MAS) breeding challenging [33]. Due to the low recombination rate and limited allelic variation in mapping populations, the precision of QTL mapping is often low, necessitating further in-depth research into the genetic basis of wheat protein quality traits. Unlike linkage mapping, genome-wide association studies (GWAS) can fully utilize recombination information in natural populations, offering high-resolution genetic variation analysis and enabling precise mapping of quantitative traits. In addition, the development of the 90K, 660K, and 100K SNP wheat arrays has made it feasible to genotype large germplasm collections with high-density SNPs. Thus, GWAS have been widely used to investigate allelic diversity for complex traits in crops like rice, maize, and wheat [34,35,36].

GPC and WGC are critical factors determining wheat processing quality [8,10,31]. In this study, 310 common wheat accessions, mainly from China and Europe, were collected, and genome-wide array data were used to evaluate the GPC and WGC across multiple environments (Table A1). Our objectives were to (1) identify key genetic loci controlling GPC and WGC; (2) develop KASP markers for high-quality breeding to accelerate the wheat breeding process; and (3) identify the candidate genes for future wheat quality study.

## 2. Results

### 2.1. Phenotypic Evaluation

**Figure 1:**
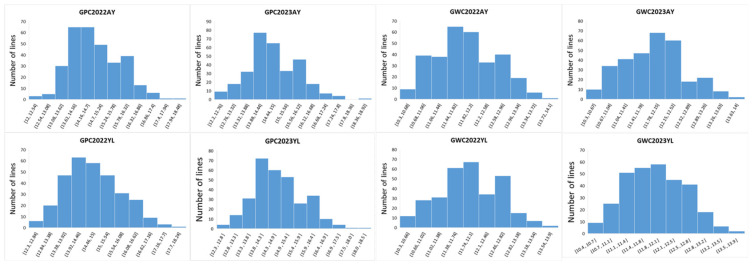
The distribution of the GPC and WGC in the diverse panel. GPC, grain protein content; WGC, wet grain content; AY (Anyang, Henan); YL (Yangling, Shaanxi); 2022/2023 indicate the harvest years.

We measured the GPC and WGC of 310 wheat varieties across four environments. Both WGC and GPC exhibited continuous variation in all environments. Across different environments, the mean values of GPC ranged from 12.3% to 18.1%, with an average of 14.7%. The standard deviation for GPC was 1.0%, and the coefficient of variation was 6.8%. Meanwhile, the mean values of WGC ranged from 10.4% to 13.6%, with an average of 11.9%. The standard deviation for WGC was 0.68%, and the coefficient of variation was 5.7% (Table 1; Figure 1). All environments for GPC and WGC showed no significant deviation from normality. The *p*-values for the JB test were all greater than 0.05 (range: 0.720–0.915). Therefore, the data can be considered normally distributed and are suitable for subsequent parametric statistical analyses (Table A2). These results indicate significant phenotypic diversity in both GPC and WGC. This diversity makes the population ideal for conducting GWAS. ANOVA across multiple environments revealed highly significant effects (*p* ≤ 0.001) of genotype (G), environment, and genotype × environment (G × E) interactions on both GPC and WGC (Table A3). The broad-sense heritability (*H*_b_^2^) estimates for GPC and WGC were 0.65 and 0.62, respectively, suggesting that phenotypic variation is primarily driven by genetic factors and thus suitable for further association mapping studies.

### 2.2. Genotyping and Population Structure Analysis

**Figure 2:**
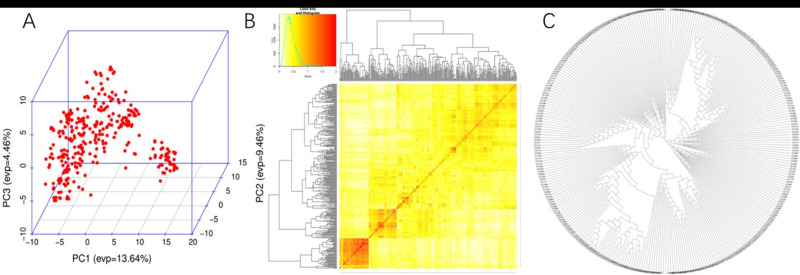
The (**A**) PCA, (**B**) NJ-tree, and (**C**) kinship for the 310 common wheat accessions.

The 100K SNP array based on the consensus genetic maps and physical map (IWGSC, http://www.wheatgenome.org/) was chosen for GWAS. After removing the SNPs with MAF (minor allele frequency) < 5% and missing data > 20%, 108,836 SNPs were employed for subsequent analysis (Table A3). These markers spanned a physical distance of 14,223.3 Mb, with an average density of 7.7 markers per Mb. A total of 38,910, 38,923, and 31,003 markers were from the A, B, and D genomes, respectively, with corresponding map lengths of 4974.7, 5248.0, and 4000.6 Mb. The marker density for the B genome (7.4 markers per Mb) was lower than that for the A (7.8 markers per Mb) and D (7.7 markers per Mb) genomes. The 310 wheat accessions could be divided into four subgroups by PCA and NJ-tree analysis. Subgroup 1 consisted of 104 accessions, mainly from Henan and Shandong, including the Luomai, Xinong, Zhengmai, and Zhumai series; Subgroup 2 consisted of 89 wheat accessions from the southern Henan and Anhui provinces, which mainly included the Anke, Huacheng, Xinmai and Zhengmai series; Subgroup 3 included 67 wheat accessions from northern Henan and Shaanxi, which included the Xinong, Zhengmai and Zhongyu series; Subgroup 4 included 50 wheat accessions from Europe. The linkage disequilibrium (LD) decay distance in the Huang-Huai wheat region is approximately 8 Mb, confirming that marker densities are sufficient for reliable association analysis [37] (Table A1, Figure 2).

### 2.3. GWAS for Grain Protein Content and Wet Gluten Content

**Figure 3:**
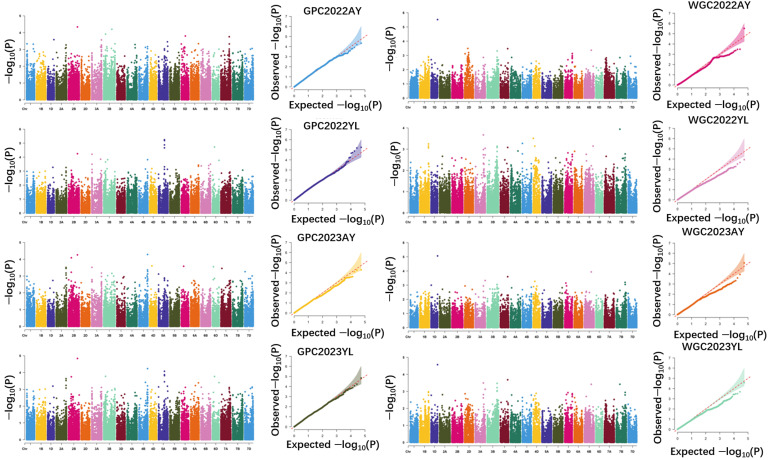
Manhattan and Q-Q plot for GPC and GWC in the diverse panel. GPC, grain protein content; WGC, wet grain content.

GWAS using 310 wheat accessions was employed to explore the genetic basis of GPC and WGC. In total, four loci for GPC and three loci for WGC were identified consistently across two or more environments. Specifically, four QTL associated with GPC were mapped on chromosomes 1A, 3A, 3B, and 4B. Notably, *QGPC.zaas-1AS* was located at 32.3 Mb on chromosome 1A and explained 8.2–8.4% of the phenotypic variation (PVE), while *QGPC.zaas-3AL* was located at 725.3–732.1 Mb on chromosome 3A and contributed 7.3–7.4% of the PVE. Additionally, *QGPC.zaas-3BL* was located at 751.8 Mb of chromosome 3B, and *QGPC.zaas-4BL* was located at 650.0 Mb of chromosome 4B, explaining 7.1–7.8% and 6.0–6.1% of the PVE, respectively. For WGC, three QTL were identified on chromosomes 4B, 5A, and 5D. Among these, *QWGC.zaas-4BL*, located at 623.8 Mb on chromosome 4B, exhibited 7.0%–10.0% of the PVEs; *QWGC.zaas-5A*, at 446.0 Mb of chromosome 5A, explained 7.4–7.6% of the PVEs; and *QWGC.zaas-5D*, at 34.6 Mb of chromosome 5D, explained 7.6–7.7% of the PVEs (Table 2; Figure 3).

### 2.4. Candidate Gene Identification

**Figure 4:**
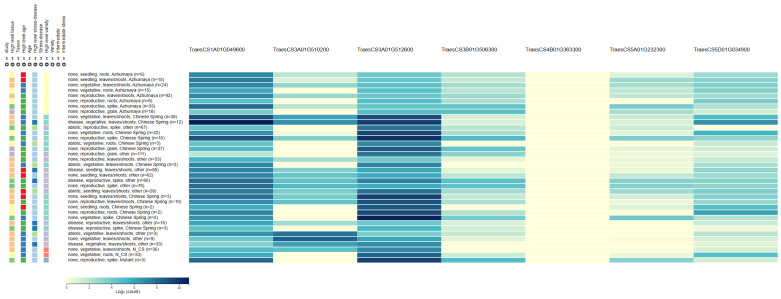
Expression pattern for the candidate genes from public database (http://wheat-expression.com/).

In total, seven candidate genes for GPC and WGC were identified based on gene annotation and expression pattern (Figure 4). For GPC, four candidate genes were identified: *TraesCS1A01G049600* for *QGPC.zaas-1AL*, located on chromosome 1A (31.3 Mb), encodes an ABC transporter; *TraesCS3A01G510200* for *QGPC.zaas-3AL*, on chromosome 3A (730.3 Mb), encodes an abscisic stress ripening protein; *TraesCS3A01G512600* for *QGPC.zaas-3AL*, on chromosome 3A (732.2 Mb), encodes a serine/threonine-protein kinase; and *TraesCS3B01G506300* for *QGPC.zaas-3BL*, on chromosome 3B (750.0 Mb), encodes an F-box family protein. For WGC, both the candidate genes *TraesCS4B01G363300* for *QWGC.zaas-4BL* on chromosome 4B (652.2 Mb) and *TraesCS5A01G232300* for *QWGC.zaas-5A* on chromosome 5A (447.5 Mb) encode an F-box family protein, whereas *TraesCS5D01G034900* on chromosome 5D (34.1 Mb) for *QWGC.zaas-5DS* encodes a UDP-glycosyltransferase (Table 3).

### 2.5. Five Breeding Available KASP Markers Were Developed and Validation

**Figure 5:**
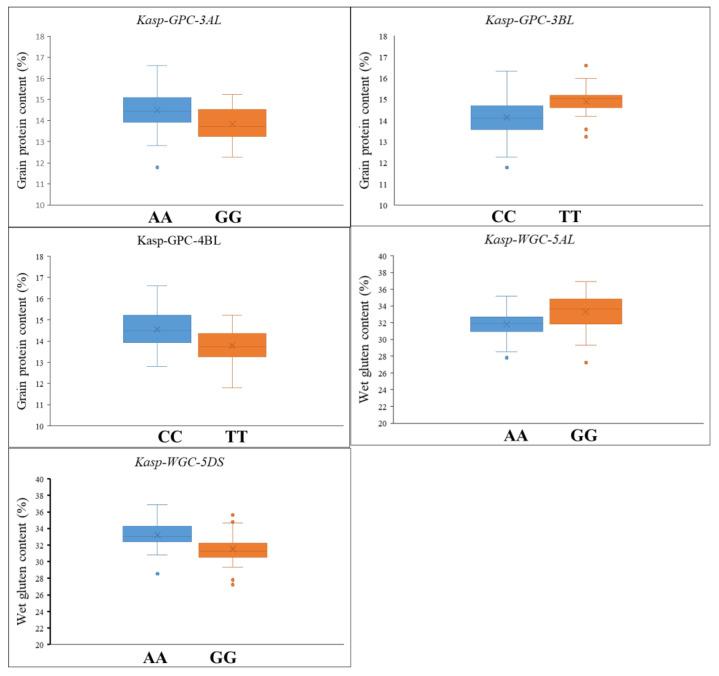
The KASP markers validated in another 123 diverse panel. GPC, grain protein content; WGC, wet grain content.

All stable loci for GPC and WGC were employed in the development of KASP markers. Another diverse panel of 123 cultivars was used to validate the efficacy of the KASP markers. A total of five KASP markers—*Kasp-GPC-3AL* for *QGPC.zaas-3AL* at 732.8 Mb of chromosome 3A; *Kasp-GPC-3BL* for *QGPC.zaas-3BL* at 750.1 Mb of chromosome 3B; *Kasp-GPC-4BL* for *QGPC.zaas-4BL* at 649.6 Mb of chromosome 4B; *Kasp-WGC-5A* for *QWGC.zaas-5A* at 444.3 Mb of chromosome 5A; and *Kasp-WGC-5DS* for *QWGC.zaas-5D* at 31.2 Mb of chromosome 5D—were successfully developed and validated (Table 4). For *Kasp-GPC-3AL*, the favorable allele (AA) accounted for 68.3% (mean GPC: 14.5%) and exhibited higher GPC compared to the unfavorable allele (GG), which accounted for 29.3% with mean GPC 13.8% (*p* < 0.05). For *Kasp-GPC-3BL*, the favorable allele (TT) accounted for 30.1% (mean GPC: 14.9%) and exhibited higher GPC compared to the unfavorable allele (CC), which accounted for 32.4%, with mean GPC of 14.1% (*p* < 0.05). For *Kasp-GPC-4BL*, the unfavorable allele (TT) accounted for 25.2% (mean GPC: 13.8%) and exhibited lower GPC compared to the favorable allele (CC), which accounted for 49.5%, with a mean GPC of 14.5% (*p* < 0.05). For *Kasp-WGC-5A*, the unfavorable allele (AA with 82 lines, mean WGC 31.8) showed lower WGC than the favorable allele (GG with 41 lines, mean WGC 33.3) (*p* < 0.05). For *Kasp-WGC-5DS*, the favorable allele (AA with 45 lines, mean WGC of 33.2) showed higher WGC than the unfavorable allele (GG with 47 lines, mean WGC of 31.5) (*p* < 0.05) (Table 5 and Table A5; Figure 5).

## 3. Discussion

The processing quality of grains directly determines the industrial value of flour and the market competitiveness of end-use products [2]. Research on wheat processing quality is of great strategic significance for ensuring food security and enhancing the added value of agricultural products [20,21,22]. The formation of wheat processing quality is a complex genotype-by-environment (G × E) interaction process: environmental factors (e.g., temperature, moisture, and nitrogen supply) significantly modulate GPC and WGC [30,32], whereas genotype defines the upper limit of genetic potential for quality traits. Achieving stable superior quality traits across diverse environments remains a longstanding bottleneck in breeding practice [2].

This study utilizes germplasm primarily from the Huang-Huai wheat region of China, complemented by European varieties. The Huang-Huai region is the paramount wheat production zone in China, accounting for over 60% of the nation’s total output. It serves as a critical hub for national food security. Within this mega-region, the Henan and Shaanxi provinces are particularly emblematic. Henan consistently ranks as China’s top wheat-producing province, contributing to approximately 25–30% of the national harvest. Its vast, fertile plains and intensive cultivation practices make it an ideal representative of high-yield potential environments. In contrast, Shaanxi, spanning parts of the Guanzhong Plain and the Loess Plateau, offers greater ecological diversity, including variable rainfall and temperature gradients. Evaluating genotypes across these two distinct sub-environments (Anyang, Henan and Yangling, Shaanxi) effectively captures the significant Genotype × Environment (G × E) interactions that characterize complex polygenic traits like grain protein content, thereby enhancing the identification of stable, broadly adaptive loci. The inclusion of European wheat germplasm is equally vital for a comprehensive genetic dissection of quality traits. European wheat breeding has a longstanding and intense focus on superior processing quality, especially for bread-making. This has led to the development of elite varieties renowned for their optimal balance of protein quality and strong, stable dough properties. European germplasms often serve as international benchmarks for bread wheat quality. By integrating these accessions into our association panel, we introduce a distinct and valuable reservoir of allelic diversity for quality-related genes that may be less frequent or absent in Chinese breeding pools. This not only increases the power to detect key loci but also allows for direct allelic comparison and the identification of potentially novel, high-value variants. Thus, the combined use of regionally adapted Chinese germplasm and quality-specialized European lines creates a powerful, globally relevant genetic resource for uncovering the genetic architecture of wheat processing quality.

As an efficient genome-wide genetic dissection tool, GWAS overcomes the limitations of traditional approaches by systematically dissecting the genetic architecture of wheat processing quality at the genome-wide level. Specifically, it not only enables precise mapping of SNP loci for quality traits (e.g., GPC and WGC) but also facilitates the screening of potential candidate genes [8,10,31]. In this study, we elucidate the molecular basis underlying quality traits in wheat and provide validated markers for high-quality breeding by GWAS. The 310 wheat accessions could be divided into four subgroups, strongly correlated with their geographic origins and pedigrees. Subgroup 1 mainly consists of accessions from Henan and Shandong (e.g., Zhengmai, Zhoumai and Zhoumai series) with consistent genetic backgrounds. Subgroup 2 consists of 89 germplasm resources, with its core comprising varieties from the Henan (e.g., Zhengmai and Xinmai series) and Anhui provinces (e.g., Anke, Huacheng), and their pedigrees can be traced back to common ancestors such as Zhou 8425B. Subgroup 3 is centered on germplasm from Henan (Zhengmai and Zhongyu Series) and Shaanxi (e.g., Xinong series). The Subgroup 4 accessions all originated from Europe. The LD decay distance in the Huang-Huai wheat region is approximately 5–8 Mb, confirming that marker densities are sufficient for reliable association analysis [37].

### 3.1. Loci for Grain Protein Content

GPC and WGC are critical determinants of the nutritional and processing quality of wheat flour, making them a major focus in wheat genetic improvement. As a typical quantitative trait, GPC is controlled by multiple minor-effect genes, and its genetic basis has been extensively studied through QTL mapping and GWAS, and is mainly distributed on chromosomes 1A, 1B, 2D, 4B, 5A, 6B, 7A, and 7B [12,38,39,40,41,42,43,44,45]. Echeverry-Solarte et al. [46] reported 11 GPC QTL in a RIL population, including two major loci explaining 16.5% and 16.9% of the PVE. Similarly, Fatiukha et al. [47] mapped 12 GPC QTL, including four stable loci on 4BS, 5AS, 6BS (e.g., *Gpc-B1*), and 7BL. Jiang et al. [48] reported 17 GPC QTL, explaining 3.94–9.21% of the PVE. Luo et al. [49] identified ten stable GPC loci in 486 diverse cultivars, one of which was detected under both normal and late sowing conditions. Suliman et al. [50] conducted a GWAS in 189 spring wheat accessions and identified 37 significant SNPs associated with GPC.

The synthesis of seed storage proteins (SSPs), which is regulated at the transcriptional level, involves genes such as *TaSPR-A*, *TaSPR-B*, and *TaSPR-D*, significantly influencing GPC [51]. *Gpc-B1* remains a major and stably expressed QTL [39,52]. A recent meta-analysis by Saini et al. [53] consolidated 459 GPC-related QTL from 48 reports into 57 meta-QTL (MQTL) and seven hotspots. Li et al. [15] identified 26 QTL for GPC, with the major locus *QGPC.sau-4A.1* on chromosome 4A (636.55–662.38 Mb), explaining 16.5% of the PVE. Guo et al. [3] mapped three loci on chromosomes 3B, 7A, and 7B, while Kartseva et al. [54] identified two QTL on 5A (488.0 Mb) and 7A (694.0 Mb) in a diverse panel. Nigro et al. [55] reported stable QTL on 3B and 5A for GPC in durum wheat.

In this study, we identified four GPC loci: *QGPC.zaas-1AS* (1A, 32.3 Mb), *QGPC.zaas-3AL* (3A, 725.3–732.1 Mb), *QGPC.zaas-3BL* (3B, 751.8 Mb), and *QGPC.zaas-4BL* (4B, 650.0 Mb). Among these, *QGPC.zaas-1AS* (32.3 Mb) was proximal to the locus on chromosome 1A (25.8–36.3 Mb) reported by Kumar et al. [39], and *QGPC.zaas-3BL* (751.8 Mb) was near loci on chromosome 3B reported by Nigro et al. [55] (732.5–760.2 Mb) and Guo et al. [3] (742.5–769.3 Mb). In contrast, *QGPC.zaas-3AL* and *QGPC.zaas-4BL* showed no nearby or overlapping loci according to the reference genome, suggesting they may represent novel GPC loci.

### 3.2. Loci for Wet Gluten Content

WGC is another key quality trait that has garnered considerable research attention. The majority of wheat grain protein is gluten protein, accounting for approximately 80% of the total protein content. Gluten is composed of a network of high- and low-molecular-weight (HMW and LMW) glutenins and monomeric gliadins [56]. The HMW glutenin subunits are encoded by *Glu-A1*, *Glu-B1*, and *Glu-D1*, located on chromosomes 1AL, 1BL, and 1DL, respectively, while the LMW glutenin subunits are encoded by *Glu-A3*, *Glu-B3*, and *Glu-D3* on chromosomes 1AS, 1BS, and 1DS, respectively [40,57]. Wheat prolamins are encoded by multiple loci on group 1 and group 6 chromosomes [58]. QTL associated with WGC have been identified on chromosomes 2A, 2B, 2D, 3A, 4B, 5A, 5D, 6A, and 7A [9,12,34,40,41,45,59,60]. Li et al. [61] identified a major WGC QTL on chromosome 5A, explaining 36.0% of the PVE. Goel et al. [62] identified a QTL for WGC on chromosome 5D, accounting for 13.5% of the PVE. Lou et al. [60] mapped 9 WGC QTL on chromosomes 1A, 1B, 3A, 3D, and 6A, explaining 2.68–3.98% of the PVE. Pu et al. [12] reported a QTL, *QWgc.sicau-5DL* by GWAS, explaining 5.0–7.9% of the PVE. In addition, Li et al. [15] reported 14 QTL for WGC, explaining 5.65–12.6% of the PVE. Zhao et al. [8] identified 4 QTL distributed on chromosomes 1B, 3D, 4B, and 5D, explaining 6.36–13.69% of the PVE. In this study, three loci for WGC were identified: *QWGC.zaas-4BL* on chromosome 4B (623.8 Mb), *QWGC.zaas-5A* on chromosome 5A (446.0 Mb), and *QWGC.zaas-5D* on chromosome 5D (34.6 Mb). No previously reported WGC loci were located near *QWGC.zaas-4BL* and *QWGC.zaas-5A*, whereas *QWGC.zaas-5D* (34.6 Mb) was near the loci reported by Zhao et al. [8] (25.6–32.1 Mb) and Goel et al. [62] (33.6–39.8 Mb). Thus, *QWGC.zaas-4BL* and *QWGC.zaas-5A* are likely novel WGC loci.

### 3.3. The Candidate Genes for Grain Protein Content and Wet Gluten Content

The identification of candidate genes linked to GPC and WGC provides valuable insights into the molecular basis. Candidate gene *TraesCS1A01G049600* for *QGPC.zaas-1AS* encodes the ABC transporter G family member, which plays a crucial role in nutrient transport and accumulation and could directly influence GPC [63,64]. Similarly, *TraesCS3A01G510200* for *QGPC.zaas-3AL* encodes the abscisic stress ripening (ASR) protein [65,66,67], whereas *TraesCS3A01G512600* for *QGPC.zaas-3AL* encodes the serine/threonine-protein kinase. Both abscisic stress ripening protein and serine/threonine-protein kinase are involved in stress response and signaling pathways, indirectly affecting protein synthesis and storage during grain development [45,54,68,69]. The role of *TraesCS3A01G510200*, which encodes an ASR protein that belongs to the late embryogenesis abundant (LEA) family often associated with drought tolerance, is pivotal in normal seed development. During grain filling, a developmental surge in abscisic acid (ABA) acts as a central regulator, orchestrating nitrogen remobilization and triggering the expression of LEA proteins like ASR. In this context, ASR proteins transition from stress protectants to essential developmental stabilizers. As the endosperm undergoes natural dehydration, ASR proteins function as molecular chaperones, safeguarding newly synthesized gluten proteins (gliadins and glutenins) from misfolding or aggregation, thereby directly influencing final protein composition and gluten network integrity. The F-box family proteins (*TraesCS3B01G506300* for *QGPC.zaas-3BL*, *TraesCS4B01G363300* for *QWGC.zaas-4BL*, and *TraesCS5A01G232300* for *QWGC.zaas-5A*) are known to participate in protein degradation and regulatory processes and could modulate the composition and quality of gluten proteins [10,70,71]. Additionally, the UDP-glycosyltransferase (*TraesCS5D01G034900* for *QWGC.zaas-5D*) plays a role in carbohydrate metabolism, influencing the interaction between grain protein and starch components [72,73,74]. These candidate genes not only enhance our understanding of the genetic basis of GPC and WGC but also provide potential targets for MAS and gene editing in wheat breeding programs. Future functional validation of these genes will be essential to confirm their roles and explore their potential for improving wheat quality traits.

### 3.4. The Validation of the KASP Markers for Grain Protein Content and Wet Gluten Content

The application of high-throughput KASP technology has significantly accelerated the wheat breeding process, particularly by enabling rapid screening of superior germplasm in early generations [1,30,75,76]. Compared to traditional phenotype-based selection methods, the KASP platform effectively overcomes their limitations of being time-consuming and inefficient [1]. KASP technology bridges the gap between high-resolution discovery and high-throughput, cost-effective application in breeding. It translates tightly linked or significantly associated markers into practical, breeder-friendly tools. Although conventional breeding methods have also played a crucial role in improving wheat processing quality, the integration of MAS with traditional techniques substantially enhances breeding efficiency, providing more precise and efficient technical support for breeding efforts. By developing and validating KASP markers for the stable loci identified, we directly provide the wheat breeding community with ready-to-use, efficient molecular tools for accelerating the improvement of grain quality traits through MAS. The loci that exhibit stability across multiple environments, such as the *QGPC.zaas-4BL* for wheat GPC and corresponding KASP marker *Kasp-GPC-4BL*, as well as the *QWGC.zaas-5D* locus for WGC and corresponding KASP marker *Kasp-WGC-5DS*, are prime candidate loci for MAS breeding. Additionally, some loci, although previously reported, have been further validated, confirming their reliability and widespread presence in polymorphic germplasm. These loci can also serve as important candidates for wheat MAS breeding. In natural populations, wheat varieties carrying favorable alleles with better grain quality traits can be utilized as parental materials to enhance processing quality. By leveraging these molecular markers and elite germplasm, breeders can expedite the development of wheat varieties with improved quality traits, thereby contributing to the long-term progress of the wheat industry.

GPC and WGC are crucial determinants of wheat processing quality, rendering their genetic improvement a primary focus in global wheat breeding research [77]. In recent years, the rapid progress in genomics and biotechnology has given rise to three remarkable trends [53,78]. Firstly, high-throughput molecular marker technologies, including GWAS and QTL mapping, have identified numerous critical genetic loci governing GPC and WGC across diverse wheat germplasm resources. Secondly, gene-editing technologies, such as CRISPR/Cas9, offer novel approaches for the precise enhancement of quality traits. Thirdly, multi-trait integrated breeding strategies are attracting growing attention, with the aim of integrating high yield, stress resistance, and superior quality to develop elite varieties adaptable to diverse environments. In the future, wheat quality breeding will increasingly depend on the integration of multi-omics data, utilizing artificial intelligence algorithms to predict optimal allele combinations and incorporating rapid breeding techniques to shorten breeding cycles [1]. Moreover, climate-resilient breeding will surface as a new priority, especially in investigating the impacts of drought stress on grain protein formation. These advancements will offer crucial scientific and technological support to the global wheat industry in tackling food security challenges and meeting evolving market demands.

## 4. Materials and Methods

### 4.1. Plant Materials and Field Trials

In this study, 310 elite wheat varieties used for GWAS and 123 varieties used to validate the KASP markers’ effect were selected (Table A1). Specifically, 258 wheat varieties were primarily from the Huang-Huai wheat region of China, including released varieties and advanced generation lines, with main source provinces including Henan, Shandong, Anhui, Jiangsu, and Shaanxi. Additionally, 52 varieties are major promoted wheat varieties and advanced generation lines from Europe, which have been screened for over two years to ensure they can mature normally in regions such as Henan, Shaanxi in China.

Field experiments were conducted over two consecutive growing seasons (2022–2023 and 2023–2024) at Anyang in Henan (114.02° E, 32.59° N) and Yangling in Shaanxi (108.04° E, 34.70° N). The experimental fields had moderate and uniform fertility, and the trials were arranged in a randomized complete block design with three replications. Each plot consisted of three 2.0 m long rows with a row spacing of 0.25 m and a plant spacing of 10 cm. Seeds were manually sown using the single-seed dibbling method, and field management followed local high-yield cultivation practices. After harvest, the grains were naturally sun-dried and stored. Impurities and damaged kernels were removed, and grain protein content and wet gluten content were measured using a Swiss-made near-infrared quality analyzer (DA7200).

### 4.2. Genotyping and Population Structure Analysis

The 310 wheat accessions were genotyped using the wheat 100K SNP array provided by Molbreeding (https://www.molbreeding.com/, Shijiazhuang, China). After obtaining the raw genotype data, quality control was performed, and SNPs with missing rates > 20% and minor allele frequency (MAF) < 0.05 were filtered out. SNPs meeting the quality control criteria were used for subsequent association analysis. The physical positions of these markers were determined by aligning their flanking sequences to the reference genome IWGSC v2.1 using BLAST. Principal component analysis (PCA) and neighbor-joining tree (NJ-tree) construction were conducted using Tassel V5.0.

### 4.3. Phenotyping Analysis

The GPC and WGC of 310 wheat varieties were measured across four environments over two years. Data analysis was performed using SAS Windows 9.2 (http://www.sas.com). Analysis of variance (ANOVA) for GPC and WGC was conducted using the PROC GLM procedure, while the correlation analysis between GPC and WGC was performed using the PROC CORR procedure. Broad-sense heritability (*H*_b_^2^) was calculated using the formula: *H*_b_^2^ = σ_g_^2^/(σ_g_^2^ + σ_ge_^2^/r + σε^2^/re), where σ_g_^2^, σ_ge_^2^, and σ_ε_^2^ represent the variance estimates for genotype (variety), genotype-by-environment interaction, and error, respectively; e and r denote the number of environments and replicates per environment, respectively. The correlation between GPC, WGC, and the developed functional markers was validated using the GPC and WGC from the four environments and their mean values. Differences in GPC and WGC among different genotypes were tested using the T-test method. The normality of each environment for GPC and WGC were assessed using the Jarque–Bera (JB) test in Excel. The test evaluates whether a dataset’s skewness and kurtosis match those of a normal distribution.

### 4.4. Association Analysis and Candidate Gene Identification

PCA and phylogenetic tree analysis revealed significant population structure among the 310 common wheat accessions, which could lead to false-positive association results. Therefore, a mixed linear model (MLM, PCA + K model) in Tassel v5.0 was used for GWAS analysis. The kinship matrix and PCA were both generated using Tassel v5.0. After Bonferroni-Holm multiple testing correction (α = 0.05), markers with an adjusted −log_10_(*p*-value) ≥ 3.0 were considered significant marker-trait associations (MTAs). Based on the *p*-values obtained from Tassel V5.0, Manhattan and Q-Q plots were generated using the CMplot package (https://github.com/YinLiLin/CMplot, accessed on 12 June 2025; R 3.6.5). To identify candidate genes within QTL regions, annotated genes located within ±3.0 Mb of the peak SNP physical position were extracted from IWGSC v1.0. Hypothetical proteins, transposon-related proteins, and retrotransposon-associated proteins were excluded to preliminarily determine candidate genes. To investigate the expression patterns and identify candidate genes with notable transcription levels in seedlings or root tissues, we utilized the publicly accessible *Triticum aestivum* gene expression database (http://wheat-expression.com/, accessed on 15 June 2025).

### 4.5. KASP Marker Development and Validation

SNP markers tightly linked to stable QTL with large effects across multiple environments were converted into KASP markers [75]. Primer design was performed using the PolyMarker online tool (http://www.polymarker.info/, accessed at 25 June 2025). KASP markers typically consist of three primers: two allele-specific primers, labeled with FAM (3′-GAAGGTGACCAAGTTCATGCT-5′) and HEX (3′-GAAGGTCGGAGTCAACGGATT-5′) fluorescent dyes, respectively, and one common primer. The PCR reaction was performed in a 4 µL volume, containing 0.048 µL primer mix, 2.0 µL MTsAer Mix, and 1.952 µL template DNA (50 ng/µL). The primer mix consisted of 12% HEX primer (forward primer 2), 12% FAM primer (forward primer 1), and 30% common primer (reverse primer), all synthesized by Sangon Biotech (Shanghai, China). Amplification was carried out on a 384-well PCR instrument (AIO-RTG, S1000AMAhermTl GyGler) with the following program: 94 °C for 15 min; 10 cycles of 94 °C for 20 s, 63–55 °C for 1 min (decreasing 1 °C per cycle); and 32 cycles of 94 °C for 20 s, 55 °C for 60 s. After completion, fluorescence data were read on a PHERTsATrplus SNP plate reader (AMG LTAAEGH) and analyzed using KlusAerGTller v3.4 (LGG, HoGGesGon, UK) to obtain genotype data.

## 5. Conclusions

This study promotes understanding of the genetic basis of wheat processing quality by identifying stable loci associated with GPC and WGC in 310 wheat varieties. In total, four loci are novel, whereas the other three loci are near known QTL or overlap with them. Moreover, seven candidate genes encoding key enzymes were identified, providing novel perspectives on the regulation of quality traits. The development of KASP markers for stable loci facilitates efficient MAS, which expedites the breeding process for enhancing wheat quality. These findings offer valuable genetic resources and practical tools for improving elite wheat germplasm. Future research should concentrate on the functional verification of candidate genes and optimization of MAS strategies to further promote wheat quality improvement.

## Figures and Tables

**Table 1:** Summary of the GPC and WGC in the 310 winter wheat accessions.

	GPC ^a^ (%)				WGC ^b^ (%)			
	2022AY ^c^	2023AY	2022YL	2023YL	2022AY	2023AY	2022YL	2023YL
Max	18.4	18.4	18.1	18.1	13.8	14.0	13.7	13.8
Min	12.0	12.2	12.3	12.2	10.3	10.3	10.3	10.3
Average	14.7	14.7	14.7	14.7	11.9	11.9	11.9	11.9
Standard deviation	1.04	1.09	1.05	1.06	0.73	0.71	0.70	0.72
Coefficient of variation	0.070	0.074	0.071	0.070	0.062	0.060	0.059	0.060

^a^ GPC, grain protein content; ^b^ WGC, wet grain content; ^c^ 2022AY: 2022Anyang; 2023AY: 2023Anyang; 2022YL: 2022Yangling; 2023YL: 2023Yangling.

**Table 2:** The loci for GPC and WGC identified in the diverse panel by GWAS.

Name ^a^	Environment	Chr. ^b^	Position (Mb)	*p*-Value	R^2^ (%)
*QGPC.zaas-1AS*	E1, E2	1A	32.3–32.3	2.8 × 10^−4^~3.0 × 10^−4^	8.2–8.4
*QGPC.zaas-3AL*	E1, E2	3A	725.3–732.1	6.6 × 10^−4^~6.8 × 10^−4^	7.3–7.4
*QGPC.zaas-3BL*	E2, E2	3B	751.8–751.8	8.3 × 10^−4^~8.3 × 10^−4^	7.1–7.8
*QGPC.zaas-4BL*	E1, E2	4B	650.0–650.0	5.1 × 10^−4^~5.7 × 10^−4^	6.0–6.1
*QWGC.zaas-4BL*	E2, E2, E3	4B	623.8–623.8	5.1 × 10^−5^~9.2 × 10^−4^	7.0–10.0
*QWGC.zaas-5A*	E1, E2	5A	446.0–446.0	5.5 × 10^−4^~5.7 × 10^−4^	7.4–7.6
*QWGC.zaas-5DS*	E1, E3	5D	34.6–34.6	4.7 × 10^−4^~5.0 × 10^−4^	7.6–7.7

^a^ GPC, grain protein content; WGC, wet grain content; ^b^ Chr.: Chromosome.

**Table 3:** The candidate genes for the loci of GPC and WGC identified in the diverse panel by GWAS.

QTL ^a^	Candidate	Chr. ^b^	Position (Mb)	Annotation
*QGPC.zaas-1AL*	*TraesCS1A01G049600*	1A	31.3	ABC transporter family member
*QGPC.zaas-3AL*	*TraesCS3A01G510200*	3A	730.3	Abscisic stress ripening protein
*QGPC.zaas-3AL*	*TraesCS3A01G512600*	3A	732.2	Serine/threonine-protein kinase
*QGPC.zaas-3BL*	*TraesCS3B01G506300*	3B	750.0	F-box family protein
*QWGC.zaas-4BL*	*TraesCS4B01G363300*	4B	652.2	F-box family protein
*QWGC.zaas-5A*	*TraesCS5A01G232300*	5A	447.5	F-box family protein
*QWGC.zaas-5DS*	*TraesCS5D01G034900*	5D	34.1	UDP-glycosyltransferase

^a^ GPC, grain protein content; WGC, wet grain content; ^b^ Chr.: Chromosome.

**Table 4:** The primers of the KASP markers developed in this study.

Kasp	QTL	Position (bp)	FAM	HEX	Common
*Kasp-GPC-3AL*	*QGPC.zaas-3AL*	732,779,582	cagttcaaagtgggatgttaaatgA	cagttcaaagtgggatgttaaatgG	ccttgatatgcatacatctggAaaT
*Kasp-GPC-3BL*	*QGPC.zaas-3BL*	750,139,050	TatcatagtagcaccacgcacgT	TatcatagtagcaccacgcacgC	atgaagcagagAggggaaTgaTaG
*Kasp-GPC-4BL*	*QGPC.zaas-4BL*	649,605,374	ttattttatcagcggCtgCatcgaT	ttattttatcagcggCtgCatcgaC	gAacatcccaaatgcgaaccaaC
*Kasp-WGC-5AL*	*QWGC.zaas-5A*	444,264,937	ggtgtatgacgcCgaattcA	ggtgtatgacgcCgaattcG	ggtGatAatttccttctcCgaatG
*Kasp-WGC-5DS*	*QWGC.zaas-5DS*	31,273,513	ctcatccatcaatgacatcatttcA	ctcatccatcaatgacatcatttcG	gcgacagtcttaccgaatcttc

GPC, grain protein content; WGC, wet grain content.

**Table 5:** Five KASP markers were developed and validated in the diverse panel.

Marker	QTL	Genotype	Number of Lines	Phenotype (%)	*p* Value
*Kasp-GPC-3AL*	*QGPC.zaas-3AL*	AA	84	GPC: 14.5	*p* < 0.01
		GG	37	GPC: 13.8	
*Kasp-GPC-3BL*	*QGPC.zaas-3BL*	TT	23	GPC: 14.9	*p* < 0.01
		CC	100	GPC: 14.1	
*Kasp-GPC-4BL*	*QGPC.zaas-4BL*	TT	31	GPC: 13.8	*p* < 0.01
		CC	61	GPC: 14.5	
*Kasp-WGC-5A*	*QWGC.zaas-5A*	AA	82	WGC: 31.8	*p* < 0.01
		GG	41	WGC: 33.3	
*Kasp-WGC-5DS*	*QWGC.zaas-5D*	AA	45	WGC: 33.2	*p* < 0.01
		GG	47	WGC: 31.5	

GPC, grain protein content; WGC, wet grain content.

## Data Availability

The original contributions presented in this study are included in the article. Further inquiries can be directed to the corresponding authors.

## Abbreviations

GWAS	Genome-wide association study
KASP	Kompetitive allele-specific PCR
GPC G	Rain protein content
WGC	Wet gluten content
QTL	Quantitative trait loci
SNP	Single nucleotide polymorphism

## Appendix A

**Table A1:** The details of the GPC and GWC for the 310 accessions.

Name	Subgroup	Grain Protein Content (%)				Wet Gluten Content (%)			
		E1	E2	E3	E4	E1	E2	E3	E4
04Zhong36	3	15.1	15.8	14.7	15.2	11.9	11.1	11.2	11.4
19CA27	1	15.9	14.7	15.8	15.5	12.2	11.8	12.3	12.1
19CA97	1	15.8	16.2	16.2	16.1	12.3	12.8	12.5	12.5
Anke1702	2	13.8	14	14.5	14.1	10.9	11	11.3	11.1
Anke1704	2	15.5	15.3	14.9	15.2	12.8	12.6	12.5	12.6
Anke1901	2	13.8	13.8	14.2	13.9	12.1	11.9	12.1	12.0
Anke1908	2	12.2	12.6	12.3	12.4	11.5	11.7	11.6	11.6
Anke2006	2	13.9	14.1	14.4	14.1	13.8	12.7	13	13.2
Anke2007	2	14.3	13.3	13.1	13.6	12.8	12.3	12.7	12.6
Anke2008	2	14.3	14.3	14.6	14.4	12.8	12.2	12.6	12.5
Anke203	1	13.5	12.8	12.8	13.0	11.4	11.1	11.4	11.3
Anong1589	-	14.2	14	13.5	13.9	13.1	13.3	12.8	13.1
Anong1928	-	15	14.5	14.8	14.8	11.7	12	12.4	12.0
Bainong207	1	12.8	14	13.7	13.5	11.5	11.7	12.1	11.8
BainongChengzhu21	3	13.5	14.2	14	13.9	12	11.9	12	12.0
Baofeng1903	3	13.1	12.6	13.7	13.1	11.2	10.8	11.5	11.2
Baofeng216	2	14.3	14	13.6	14.0	13.5	13.1	12.7	13.1
Baoliang5hao	1	15.3	14.9	15.1	15.1	12	11.9	12	12.0
Changmai20	1	13.8	13.5	14.4	13.9	11.1	10.8	11.3	11.1
Cunmai16hao	3	16.4	15.8	16.8	16.3	11.7	11.7	12.2	11.9
Cunmai35	2	14.1	14.5	14.4	14.3	12.7	12.7	12.9	12.8
Dehongfumai21	3	15	15.8	15.6	15.5	11.7	12.1	11.3	11.7
Fanmai26	2	14.2	14.7	14.1	14.3	13.2	13.5	13.4	13.4
Fanyuemai32	-	14.4	14.2	13.9	14.2	12.2	12.2	11.5	12.0
FengdeCunmai16hao	1	15.5	15	14.8	15.1	12.1	12.2	12.4	12.2
Fuma916	-	15.6	14.9	15.9	15.5	12.8	12.9	12.9	12.9
Fumai16	2	14.5	14.8	14.1	14.5	10.9	11.5	11	11.1
Fumai33	1	14.9	14.2	13.3	14.1	11.8	12	12	11.9
Guanmai13	3	14.2	14.3	13.6	14.0	11.2	11.1	10.9	11.1
Hengmai175345	1	14.1	14.5	14.3	14.3	12	12	12.3	12.1
Hengmai175364	2	13.8	14.1	13.9	13.9	11	11.6	11.4	11.3
Huacheng138	2	14.3	14.8	15	14.7	12	12.4	12.1	12.2
Huacheng865	2	13.9	13.5	14.1	13.8	12.4	11.9	12.3	12.2
Huacheng9137	2	16.5	16.2	16.5	16.4	11	10.9	10.9	10.9
Huacheng916	2	16.1	14.6	15.1	15.3	12.3	11.6	11.7	11.9
Huaimai2118	3	14.7	14.3	14.4	14.5	13	13.2	12.9	13.0
Huaimai40	2	14	13.7	14.5	14.1	11.2	10.9	11.2	11.1
Huaimai404	3	15.6	16.3	16.4	16.1	11.7	11.5	11.9	11.7
Huaiming36	-	14.8	14.8	14.7	14.8	12.9	12.4	12	12.4
Huake20212	-	15.3	15	14.2	14.8	12.6	12.2	12.6	12.5
Huamai17v65	2	13.4	13.9	13.7	13.7	11.6	11.7	11.1	11.5
Huamai2003	1	13.8	14.5	13.9	14.1	10.6	11.2	10.8	10.9
Huamai2100	2	14.6	14.3	14.9	14.6	11.2	11.7	11.7	11.5
Jiamai208	1	14.9	14.8	14.3	14.7	10.6	10.9	10.4	10.6
Jimai196	2	16.3	16	16.1	16.1	12.2	11.9	12.3	12.1
Jimai20	2	16.1	15.9	16	16.0	10.6	10.6	11.3	10.8
Jimai211	1	15.2	15.6	15	15.3	12.1	11.9	12.3	12.1
Jimai418	2	14	14.5	13.8	14.1	12.3	11.2	11.9	11.8
Jìmài44	-	15.5	15	15.5	15.3	11.9	11.7	11.8	11.8
Jinhedao17278	-	13	13.4	13.5	13.3	11.1	10.8	11.4	11.1
Jiushenghe169	2	15.1	13.9	14.6	14.5	11	11.1	11.2	11.1
Jiyuanmai20	-	14	14.6	14	14.2	11.7	11.7	11.5	11.6
Kedaxia1026	-	14.9	14.4	14.4	14.6	11.3	10.7	10.8	10.9
Kedaxia116	-	14.6	14.7	14.1	14.5	11.8	11.8	11.8	11.8
Liangke6hao	2	14	13.9	13.6	13.8	13.1	12.6	13.1	12.9
Lianmai1901	2	15.4	14.8	14.2	14.8	12.1	11.7	12.4	12.1
Liuxia526	-	15	14	14.2	14.4	12.3	11.9	11.9	12.0
Longmai1hao	2	13.6	12.6	13.2	13.1	10.8	10.6	11.5	11.0
Lunxuan136	3	15.1	15.9	14.7	15.2	11.7	11.9	11.5	11.7
Lunxuan148	2	15.9	16.3	16.5	16.2	12.1	12.3	12.5	12.3
Lunxuan369	1	14.6	14.1	14.4	14.4	11.4	11.2	11.6	11.4
Luomai26	3	14.9	15.3	15.3	15.2	11.5	10.8	10.9	11.1
Luomai28	2	16.1	15.6	15.4	15.7	11.1	11.8	11.7	11.5
Luomai44	1	13.8	13.7	14.2	13.9	11	10.9	11.4	11.1
Luomai45	1	14.1	14.8	13.6	14.2	11	11.3	10.9	11.1
Luomai49	1	16.7	16.4	16.1	16.4	11.3	11	10.3	10.9
Luomai50	1	13.9	15.1	15.2	14.7	11	11.3	10.9	11.1
Luomai54	1	16.4	16.1	16.1	16.2	10.8	10.8	11.3	11.0
Luomai56	2	15.1	14.2	15.2	14.8	12.1	11.8	11.6	11.8
Luomai59	1	13.5	13.1	14.9	13.8	11.5	11.3	11.4	11.4
Luoqing1905	-	15.3	15.4	15.6	15.4	12.5	12	12.4	12.3
Luoqingfeng2101	-	13.7	14.5	13.9	14.0	10.7	11.1	10.6	10.8
Luoqingfeng7011	-	13.4	13.6	13.9	13.6	10.8	10.7	10.7	10.7
Luoqingmai109	-	13.5	13.4	13.6	13.5	11.6	11.8	11.6	11.7
Luoqingmai40	-	16.4	15.5	15.7	15.9	11.6	11.2	10.9	11.2
Luoqingmai56	-	12.6	13.2	13.2	13.0	11.5	11.9	12.1	11.8
Luoqingmai69	-	14.2	13.9	13.9	14.0	13.4	13.1	13.2	13.2
Luoqingmai70	-	15.7	14.3	14.4	14.8	10.7	10.6	10.5	10.6
Nongda759	2	14	14	14.2	14.1	12.5	12.5	12.7	12.6
Nongke1132	2	16.3	15.9	15.4	15.9	12.2	12.5	12.5	12.4
Nongxin699	2	15.8	15.7	15.7	15.7	11.9	11.6	11.7	11.7
Pingan0518	-	15.7	16	15	15.6	11.7	11.9	11.7	11.8
Pingan18	-	15.6	14.9	15.4	15.3	12.8	12.8	12.5	12.7
Puling300	-	13.6	13.9	14.1	13.9	11.1	11.6	11.4	11.4
Puling35038	-	13.8	13.7	14.2	13.9	10.8	10.3	10.5	10.5
Pumai126	1	15.2	15	15.9	15.4	11.6	12	11.6	11.7
Pumai127	1	14.1	13.5	13.3	13.6	11	12.1	11.6	11.6
Pumai157	1	15.8	15	15	15.3	12.3	12.4	12	12.2
Pumai186	1	14.5	15	15.6	15.0	11.9	11.6	11.8	11.8
Puxing26hao	3	15.5	15.4	15.7	15.5	11.5	11	11.5	11.3
Puxing35hao	1	15.3	15.1	15.5	15.3	10.8	11	10.8	10.9
Ruihua519	2	17	17.7	18.1	17.6	13.1	12.9	12.3	12.8
Ruihuamai236	3	14.2	13.5	13.8	13.8	12.9	13	12.7	12.9
Ruihuamai513	1	17.6	17.1	15.2	16.6	11.6	11.1	11.4	11.4
Saidemai216	1	13.3	13	12.6	13.0	12.2	11.5	11.7	11.8
Saidemai5hao	1	15.9	16.3	16	16.1	11.9	11.9	11.5	11.8
Sanfengmai616	3	15.4	15.2	14.8	15.1	11.4	11.4	11.3	11.4
Shangmai187	2	13.7	13.7	13.2	13.5	12.3	12.8	12.1	12.4
Shangmai198	3	13	13.5	13.1	13.2	12.6	12.8	12.5	12.6
Shangmai206	1	15.9	16	15.8	15.9	11.2	11.4	11.4	11.3
Shanhe1029	2	14.8	15.2	14.9	15.0	12.9	13	13.4	13.1
Shengmaiyuan789	1	14.1	14.9	13.7	14.2	11.6	11.3	10.6	11.2
Shi15-6375	2	14.1	13.7	13.8	13.9	13.2	12.8	12.3	12.8
Shunmai10hao	1	14.4	15.6	15.5	15.2	11	11.4	11.3	11.2
Taiheimai10hao	-	15.6	14.1	15.1	14.9	12.3	12.6	12.5	12.5
Tianmai116	1	15.7	15.9	15.2	15.6	10.8	11.1	10.5	10.8
Tianmai159	1	14.9	14.6	14.8	14.8	12	12.1	12.2	12.1
Tianmai160	1	14	14.2	14	14.1	12.9	12.8	12.6	12.8
Tianmai177	1	15.2	15.5	15	15.2	11.6	11.7	11.6	11.6
Tianmai196	1	14.1	14.2	14	14.1	11.6	11.5	10.8	11.3
Tianmin360	3	16.6	15.9	16.8	16.4	11.2	11.4	11.7	11.4
Tianning138	3	14.6	14.9	15.1	14.9	12.7	12.9	12.4	12.7
Tianyikemai15	2	15.4	14.6	14.8	14.9	12.9	12.5	12.6	12.7
Tuomai303	-	13.8	13.2	13.7	13.6	11.5	11.2	11.1	11.3
Tuomai44	-	14.4	14.2	14.7	14.4	11.7	11.4	11.8	11.6
Tuomai606	-	14.1	14.8	14.7	14.5	11.9	12.4	11.7	12.0
Tuyu16	-	13.5	13	12.8	13.1	11.2	11.6	10.8	11.2
W1	4	14.2	14.2	15.1	14.5	13	12.5	12.9	12.8
W10	4	13.4	13.8	14.1	13.8	13.1	12.3	12.8	12.7
W11	4	15.1	15.3	15.1	15.2	12.3	12.7	12.4	12.5
W12	4	15.2	14.9	14.9	15.0	12	12.2	12.1	12.1
W13	4	15	15.5	14.2	14.9	12.5	13.2	12	12.6
W14	4	13.7	13.7	14.1	13.8	11.7	12.1	12.2	12.0
W15	4	14.1	14.4	14	14.2	10.9	11.1	10.7	10.9
W16	4	14.4	13.1	13.9	13.8	12.3	12.3	11.9	12.2
W17	4	14.3	14	13.5	13.9	12	11.8	11.3	11.7
W18	4	14.8	14	14.5	14.4	11.7	11.8	11.8	11.8
W19	4	16.8	17	16.8	16.9	11.6	11.9	12.3	11.9
W2	4	14.9	15	14.9	14.9	12	11.7	11.9	11.9
W20	4	14.1	13.8	13.8	13.9	12	12.3	12.6	12.3
W21	4	13.2	13.6	13.2	13.3	12.6	12.5	12.3	12.5
W22	4	13.5	13.9	13.4	13.6	13.3	13.1	12.7	13.0
W23	4	15.8	15.9	14.9	15.5	12.8	13.2	12.8	12.9
W24	4	12	12.2	12.6	12.3	12.6	12.2	12.6	12.5
W25	1	14.2	14.1	14.5	14.3	10.9	11.2	11.1	11.1
W26	4	13.7	13.4	13	13.4	11.4	10.9	10.9	11.1
W27	4	14	15.3	14.5	14.6	11.9	12	11.6	11.8
W29	4	14.9	15.7	15.4	15.3	10.8	10.8	11.2	10.9
W3	4	13.4	13.6	13.5	13.5	12.2	12.3	12	12.2
W30	4	14.4	14.9	14.6	14.6	11.3	11.8	11.9	11.7
W31	4	14.9	14.4	14.6	14.6	11.3	11.4	11.5	11.4
W32	4	15.4	15.9	16.5	15.9	13.1	12.7	12.4	12.7
W33	4	15.6	15.6	15.4	15.5	12.2	12.4	11.9	12.2
W34	4	15.5	14.6	15.6	15.2	12.8	12.4	12.7	12.6
W35	4	16.1	15.3	15.9	15.8	13.3	13.3	13.2	13.3
W36	4	16.1	17.1	16.6	16.6	13.1	13.5	13.3	13.3
W37	4	14	13.8	14.1	14.0	12	12.3	11.8	12.0
W38	4	14.4	13.4	13.5	13.8	13.4	13.1	12.9	13.1
W39	4	14.7	15.9	13.9	14.8	12.6	13.1	12.6	12.8
W4	4	14	14.8	14.7	14.5	12.6	13.1	12.5	12.7
W40	4	14.8	14.1	14.4	14.4	11.7	11.9	11.3	11.6
W41	4	13.9	14.1	14	14.0	12.6	12.5	12.8	12.6
W43	4	13.8	13.7	14.6	14.0	12.1	11.8	12.3	12.1
W45	4	14.4	14.2	14.7	14.4	10.9	11	11.3	11.1
W47	4	16.7	16	15.1	15.9	12.4	12.1	12	12.2
W49	4	14.3	15	14.4	14.6	12	12.2	12	12.1
W5	4	14	13.2	13.1	13.4	11.7	11.6	11.9	11.7
W50	2	16.3	16	15.3	15.9	12.5	12.3	12.4	12.4
W51	4	13.4	13.7	13.3	13.5	12.3	11.8	11.7	11.9
W52	4	14	13.5	14.3	13.9	13.3	13	12.9	13.1
W53	4	14.8	13.8	13.7	14.1	12.1	11.6	11.8	11.8
W54	4	13.6	14.1	14	13.9	12.4	12.1	12.2	12.2
W55	4	13.9	14.2	13.8	14.0	11.7	11.6	11.9	11.7
W6	4	15	15	14.4	14.8	12.1	12.2	12	12.1
W8	4	14.6	14.8	15	14.8	11.6	11.8	11.7	11.7
W86	4	15.1	15.7	16.4	15.7	13.1	14	13.7	13.6
W87	4	14.1	14.4	14.3	14.3	12.7	12.3	12.1	12.4
W88	4	13.3	13.1	14.2	13.5	11.9	11.9	12.1	12.0
W9	4	14.5	14.8	14.8	14.7	11.8	11.9	12.2	12.0
Wanfeng505	2	13.9	13.4	14.1	13.8	10.8	11	11.1	11.0
Wanke2mai1720	-	13.9	14.6	14.1	14.2	11.2	11	11.4	11.2
Wanke800	2	13.7	14.3	13.5	13.8	11.6	12.3	12.2	12.0
Wansu1617	2	14.6	13.9	15.3	14.6	13	12.9	13.1	13.0
Wansu21	1	15.5	16.6	16.1	16.1	11.5	11.7	11.6	11.6
Xianmai283	3	13.1	12.7	13	12.9	13.4	13.1	12.7	13.1
Xiansheng369	1	13.9	14.4	14.2	14.2	12.1	12.3	13	12.5
Xinkemai176	2	14.7	15.1	15.1	15.0	11.6	11.7	12	11.8
Xinkemai186	2	15.8	16.5	15.7	16.0	11.7	12	11.7	11.8
Xinliang16	3	13.2	12.7	12.5	12.8	11.4	10.9	11.4	11.2
Xinmai26	3	15.1	14.8	14.9	14.9	12.1	11.8	12.1	12.0
Xinmai45	2	16.1	16.6	16.8	16.5	10.5	11.3	11.5	11.1
Xinmai52	2	13.7	14	14.1	13.9	12.2	12.4	11.9	12.2
Xinmai57	1	14.3	13.7	13.8	13.9	11.5	11.4	11.5	11.5
Xinmai58	2	16.7	15.7	15.7	16.0	12.1	12.4	12.2	12.2
Xinmai65	2	13.9	15.5	14.9	14.8	11.2	11.6	11.6	11.5
Xinmai66	2	14.5	14.2	15	14.6	10.7	10.8	10.9	10.8
Xinmai67	3	14.3	14.7	14.3	14.4	13.2	14	13.3	13.5
Xinmai72	1	14	14.6	14.9	14.5	11.5	11.7	11.8	11.7
Xinmai78	3	14.9	14.9	15.7	15.2	11	11.9	11.4	11.4
Xinong1125	3	15.9	16	16	16.0	12.2	11.7	12.6	12.2
Xinong1152	1	15.4	14.5	15.9	15.3	12.9	12.3	12.2	12.5
Xinong1155	3	14.8	14.7	15.5	15.0	12.4	12.3	12.7	12.5
Xinong1522	3	16.3	16.7	16.9	16.6	11.7	11.7	11.9	11.8
Xinong158	1	14.4	15.2	15.3	15.0	11.8	11.8	11.7	11.8
Xinong162	1	16	15.8	15.8	15.9	11.8	12.2	11.8	11.9
Xinong1668	3	14.5	14.3	15.3	14.7	12	12.3	12.1	12.1
Xinong1871	3	14	14.1	15.1	14.4	12.8	12.1	12.2	12.4
Xinong20	1	16.6	15.9	15.5	16.0	11	11.5	11.2	11.2
Xinong301	1	14.1	14.3	14	14.1	10.3	10.3	10.5	10.4
Xinong5189	3	15.1	15.4	15.4	15.3	12.8	13.3	13.3	13.1
Xinong5195	3	15.3	17.2	16.2	16.2	10.9	11.4	11	11.1
Xinong579	3	15.9	16.6	15.4	16.0	12.9	12.2	13.1	12.7
Xinong586	1	13.9	14.1	14.3	14.1	11.6	11.1	11.4	11.4
Xinong633	2	14.9	14.7	14.5	14.7	10.9	10.5	10.3	10.6
Xinong635	2	15.9	15.2	16.3	15.8	11.6	11.3	11.5	11.5
Xinong805a	1	16.2	15.4	16.5	16.0	11.5	12	12	11.8
Xinong839	1	15.5	16.6	16.7	16.3	12.7	13.2	12.8	12.9
Xinong857	2	14.6	14.8	14.6	14.7	11.5	11.5	10.8	11.3
Xinong867	1	14.6	13.9	14.5	14.3	11.4	11.7	11.4	11.5
Xinong9198	3	17	16.6	17.6	17.1	12.1	12.1	12.1	12.1
Xinong926	1	13.2	13.7	13.6	13.5	12.4	13.4	12.5	12.8
Xinong962	3	13.7	14.4	14.4	14.2	11.7	11.9	12.5	12.0
Xinong963	1	14.9	15	15.2	15.0	12.4	12.3	12.7	12.5
Xinong9799	-	15.8	15.6	16.5	16.0	11.1	11.6	11.5	11.4
Xinong9866	-	14.1	14.3	13.8	14.1	11.9	12.4	12.3	12.2
Xinshiji2hao	-	13.2	14	13.7	13.6	11.8	12.4	12.6	12.3
Xuke108	2	15.4	15.2	14.6	15.1	13.3	13.2	12.8	13.1
Xuke13	1	15.1	14.9	14.5	14.8	12.3	12.4	13	12.6
Xuke16	3	14.7	14.9	14.7	14.8	11.1	11.4	11.3	11.3
Xumai17016	2	14.6	13.1	13.8	13.8	12.4	12.4	12.5	12.4
Xumai17106	3	14.3	13.2	13.7	13.7	12	11.7	12.5	12.1
Xumai18197	2	14.7	14.3	15.2	14.7	12.5	12.1	13	12.5
Xumai2198	2	16.3	16	16.2	16.2	12.1	11.5	12.2	11.9
Yanbaol369	-	15.6	15.9	16.1	15.9	12.3	12.6	12.5	12.5
Yangmai15	2	13.6	12.8	13	13.1	12.7	12.2	12.5	12.5
Yingqiang1hao	2	16.5	16.4	16.6	16.5	12.6	12.2	11.6	12.1
Yunong168	1	15.8	15	15	15.3	11.9	12	11.8	11.9
Yunong612	1	12.7	12.5	12.9	12.7	12.2	12	12.3	12.2
Yunong7168	1	15.5	15.6	16	15.7	12.5	12.2	12.1	12.3
Yunong7928	2	14.8	14.7	13.6	14.4	13	12.6	13.1	12.9
Yunong804	3	12.3	12.8	12.9	12.7	11.4	11.7	11.5	11.5
Yunong905	3	15.2	16	15.2	15.5	11.8	12	11.3	11.7
Yunong907	2	14.7	14.7	15	14.8	11.9	11.7	11.9	11.8
Yunong912	1	15	15.2	14.4	14.9	11.6	11.6	11.9	11.7
Yunong917	3	15.3	15.7	15.8	15.6	12.5	12.7	12.7	12.6
Yunong923	1	14.3	14	13.8	14.0	11.8	11.7	11.3	11.6
Yutong120	2	16	16	15.9	16.0	11.5	11.7	11.6	11.6
Yuzhou121	2	15.4	15.4	15.2	15.3	12.3	12.7	12.6	12.5
Yuzhou136	3	16.5	16.6	15.5	16.2	12.9	12.9	12.5	12.8
Zhengda181	1	13.3	13	12.9	13.1	11.2	11.5	11.6	11.4
Zhengda201	1	14.6	13.9	13.3	13.9	12.5	12.2	12	12.2
Zhengmai113	2	16.3	15.7	16.1	16.0	12.6	12.3	12.9	12.6
Zhengmai132	1	13.4	13.7	14.2	13.8	10.9	11	10.4	10.8
Zhengmai136	2	16.2	17	17.1	16.8	11.5	11.3	11.7	11.5
Zhengmai139	2	15.5	15	15.4	15.3	11.6	11.4	11.2	11.4
Zhengmai163	2	14.1	14.2	14.3	14.2	12.9	12.6	12.5	12.7
Zhengmai1831	3	13.2	13.3	14.3	13.6	11.1	11.4	11.8	11.4
Zhengmai1905	1	13.7	12.6	13.6	13.3	12.7	12.5	12.5	12.6
Zhengmai1926	1	14.5	13.9	15.3	14.6	11.4	10.9	10.8	11.0
Zhengmai201	1	14.4	14.5	14.4	14.4	12	11.9	11.8	11.9
Zhengmai2018	3	14.9	15.1	15.6	15.2	11.4	11.4	11.5	11.4
Zhengmai21	2	13.6	14.1	13.6	13.8	11.5	11	11.3	11.3
Zhengmai216	2	13.9	15	15.2	14.7	12.1	12.9	12.8	12.6
Zhengmai22	1	15.8	15.9	16	15.9	12.3	12.4	12.6	12.4
Zhengmai23	1	14.5	13.7	13.9	14.0	12	12.4	11.8	12.1
Zhengmai29	1	18.4	18.4	17.5	18.1	11	10.4	10.7	10.7
Zhengmai366	3	15.1	15.4	15	15.2	10.6	10.6	11	10.7
Zhengmai518	2	14.5	14.3	14.2	14.3	12.3	11.8	12	12.0
Zhengmai6166	3	14.3	14	14.3	14.2	11.6	11.4	11.9	11.6
Zhengmai618	1	14.9	15	15.5	15.1	10.7	10.6	10.3	10.5
Zhengmai823	3	14.6	14.2	14.9	14.6	13.6	13.3	13.7	13.5
Zhengmai825	3	13.7	14.9	14.2	14.3	12.3	12.1	12.6	12.3
Zhengmai826	2	16	15.8	16.3	16.0	11.8	11.9	11.8	11.8
Zhengmai827	3	14.5	14.8	14.4	14.6	11.5	11.6	11.9	11.7
Zhengmai828	2	14.6	15.7	15.3	15.2	11.5	11.9	11.8	11.7
Zhengmai917	1	14.1	14.3	14.4	14.3	11.7	10.8	11.1	11.2
Zhengmai918	3	14.3	15.7	15.8	15.3	11	11.7	11.7	11.5
Zhengmai9699	1	17.2	17.3	16.7	17.1	13.4	13.5	12.8	13.2
Zhengpinmai25	1	14.1	14	13.3	13.8	12.1	11.8	11.8	11.9
Zhengshimai9170	-	14.2	14.2	13.6	14.0	11.2	11.3	10.7	11.1
Zhengyanmai182	1	13.7	13.9	13.8	13.8	12.7	12.2	12.5	12.5
Zhongmai255	2	16.1	16	16.4	16.2	10.9	10.7	10.7	10.8
Zhongmai6032	2	14.1	14.3	14	14.1	12.7	12.3	12	12.3
Zhongmai6301	2	15.2	15.7	15.3	15.4	10.7	11.1	10.9	10.9
Zhongmai698	1	14.8	15.3	15.3	15.1	10.9	11.2	11.4	11.2
Zhongnong031	2	14.5	15.5	15.3	15.1	11.7	10.9	11.2	11.3
Zhongnong539	3	13.5	13.2	13.4	13.4	12	11.6	11.6	11.7
Zhongyan270	1	13.8	13.3	14.6	13.9	11.1	10.9	11.5	11.2
Zhongyu049	3	16.1	16.3	15.6	16.0	11.2	11.4	11.2	11.3
Zhongyu1211	3	14.2	14.3	14.5	14.3	12.7	12.4	12.7	12.6
Zhongyu1220	1	14.7	15.3	15.5	15.2	12.1	11.8	11.6	11.8
Zhongyu130	3	15.8	16.2	16.2	16.1	10.5	10.6	10.6	10.6
Zhongyu149	3	14.9	14.9	14.9	14.9	12	12	12.3	12.1
Zhongyu1702	1	15	15.2	15.3	15.2	12	11.8	12	11.9
Zhongyu36	-	15	14.4	15.2	14.9	11.4	10.9	11.3	11.2
Zhongyu978	1	17.3	17.4	17.3	17.3	12.2	12.1	12	12.1
Zhongyu980	1	16.2	16	16.2	16.1	10.9	10.8	10.8	10.8
Zhongyuan26	1	17.2	16.6	16.3	16.7	12.6	12.1	12	12.2
Zhongyuanfeng1hao	1	16.4	17.8	15.8	16.7	11.6	12.1	11.8	11.8
Zhongzhimai13	1	14.8	13.9	14.6	14.4	13.2	12.4	12.8	12.8
Zhongzhimai22	3	13.9	14.1	13	13.7	10.4	11.5	10.8	10.9
Zhongzhu16	-	17.2	16.9	16.8	17.0	12.9	12.5	12.8	12.7
Zhoumai18	1	14.8	14.4	14.5	14.6	11.6	12.3	12.3	12.1
Zhoumai22	1	14.5	15.3	14.9	14.9	11.3	11.4	11.1	11.3
Zhoumai36	1	14.8	15.3	14.7	14.9	12.3	13	12.4	12.6
Zhoumai48	1	15.6	16.5	15.9	16.0	11.9	12.2	12.4	12.2
Zhoumai52hao	1	16	16.1	16.3	16.1	10.4	10.9	10.8	10.7
Zhumai562	1	13.1	14.5	13.4	13.7	11.2	11.9	11.6	11.6
Zhumai566	1	13.7	14.1	14	13.9	10.9	10.7	11.1	10.9
Zhumai567	2	14.6	15	14.4	14.7	12.2	11.8	12.1	12.0
Zhumai568	1	16.3	15.9	16	16.1	11.6	11.8	11.8	11.7
Zhumai569	3	14.7	14.3	14.2	14.4	11.9	11.8	11.8	11.8
Zhumai586	2	14.5	15.1	14.6	14.7	11	11.6	11	11.2
Zhumai591	1	13.7	13.6	14	13.8	12.3	12.1	12.7	12.4
Zhumai592	1	15.3	15.2	15.6	15.4	11.4	11.4	11.6	11.5
Zhumai596	1	14.5	14.3	14.2	14.3	12.9	12.2	12.6	12.6
Zhumai598	1	13.2	12.7	13.2	13.0	10.7	10.8	11.5	11.0
Zhumai599	3	14.1	14.4	13.6	14.0	12.6	12.5	12	12.4
Zhumai726	2	15.8	15.7	15.4	15.6	11.8	12	11.3	11.7
Zhumai762	3	14.6	15	14.3	14.6	11.3	11.6	11.9	11.6

E1: 2022Anyang; E2: 2023Anyang; E3: 2022Yangling; E4: 2023Yangling.

**Table A2:** Results of normality tests (Jarque–Bera).

Sample Group	Skewness	Kurtosis	JB Statistic	*p*-Value	Conforms to Normal Distribution?
E1	0.003	3.129	0.372	0.830	Yes
E2	0.102	3.107	0.466	0.792	Yes
E3	−0.003	3.168	0.656	0.720	Yes
E4	0.035	3.149	0.377	0.828	Yes
E5	0.097	2.970	0.359	0.836	Yes
E6	0.080	2.937	0.542	0.763	Yes
E7	−0.006	3.076	0.307	0.858	Yes
E8	0.059	3.038	0.177	0.915	Yes

**Table A3:** ANOVA of GPC and WGC for the diverse panel.

Source of Variation	Df	SS		MS		F-Value		*p*-Value	
		GPC	WGC	GPC	WGC	GPC	WGC	GPC	WGC
Genotypes	309	2304.1	4079.2	7.46	13.2	15.12	23.3	<0.01	<0.01
Environments	3	205,757.2	497,393.1	68,585.0	165,797.0	417,174.67	876,285	<0.01	<0.01
Replicates (environments)	2	0.9	2.2	0.5	1.1	0.91	1.9	0.4	0.14
GenotypeEnvironment	927	324.1	1924.0	1.1	2.1	2.13	11.0	<0.01	<0.01
Error	247								

GPC, grain protein content; WGC, wet grain content.

**Table A4:** SNP genotyping details of the 310 wheat accessions by wheat 100K SNP array.

Chromosome	No. of the Marker	Physical Length (Mb)	Marker Density (Marker/Mb)
1A	5156	598.6	8.6
2A	6537	787.7	8.3
3A	5606	754.0	7.4
4A	5297	754.2	7.0
5A	5733	713.3	8.0
6A	4905	622.6	7.9
7A	5676	744.5	7.6
1B	4777	700.3	6.8
2B	6541	812.7	8.0
3B	6173	851.9	7.2
4B	4659	673.6	6.9
5B	5908	714.8	8.3
6B	5284	731.1	7.2
7B	5581	763.7	7.3
1D	3931	498.6	7.9
2D	5270	656.4	8.0
3D	4703	619.5	7.6
4D	3568	518.2	6.9
5D	4755	569.8	8.3
6D	3725	495.3	7.5
7D	5051	642.8	7.9
A	38,910	4974.7	7.8
B	38,923	5248.0	7.4
D	31,003	4000.6	7.7
ALL	108,836	14,223.3	7.7

**Table A5:** The KASP markers validated in the diverse panel.

Name	*Kasp-GPC-3AL*	*Kasp-GPC-3BL*	*Kasp-GPC-4BL*	*Kasp-WGC-5AL*	*Kasp-WGC-5DS*	WGC (%)	GPC (%)
Huaimai20	GG	CC	CC	AA	GG	31.6	14.4
Huaimai18	GG	CC	CC	AA	AA	32.4	14.5
Lumai21	AA	CC	CC	GG	AA	34.2	15.3
Wanmai38	AA	CC	CC	GG	AA	33.8	15.5
Wennong5	AA	CC	CC	GG	AA	34.3	15.3
Jinmai61	AA	CC	CC	AA	AA	32.1	14.3
Xinong1376	AA	TT	CC	AA	AA	33.2	15.1
Yumai7	AA	TT	CC	GG	AA	35.6	16.6
Fengchan3	AA	CC	CC	AA	AA	32.7	14.1
Jimai20	GG	CC	CC	GG	AA	32.4	13.9
Lumai6	GG	CC	CC	AA	GG	29.7	13.7
Shiyou17	AA	CC	CC	GG	AA	34.4	15.5
Su0663	GG	CC	CC	AA	GG	29.7	12.9
Wanmai53	GG	CC	CC	AA	GG	31.6	14.1
Lankao23	AA	TT	CC	AA	AA	32.8	14.9
Linkang12	AA	CC	CC	GG	AA	35.8	16.1
Luyuan502	AA	CC	CC	AA	GG	31.3	14.1
Neixiang188	AA	CC	CC	AA	GG	33.7	15.9
Shi4185	GG	CC	CC	AA	GG	32.0	14.8
Shijiazhuang15	AA	CC	CC	AA	AA	32.7	14.5
Shijiazhuang8	AA	CC	CC	AA	GG	31.6	14.5
Xinong2000-7	AA	TT	CC	AA	AA	33.4	14.8
Xinong291	AA	CC	CC	AA	AA	33.3	15.4
Yumai13	GG	CC	CC	AA	AA	32.2	15.1
Yumai35	GG	TT	CC	AA	GG	32.5	15.2
Zhongmai871	GG	CC	CC	GG	GG	33.6	15.2
Zhongmai875	GG	TT	CC	GG	AA	34.3	14.7
Zhongmai895	GG	CC	CC	GG	AA	34.8	14.7
Zhongyu9	AA	TT	CC	AA	GG	30.7	14.2
Shannong20	AA	CC	CC	AA	AA	28.5	12.8
Shan150	AA	CC	CC	GG	GG	34.8	16.3
Taishan5	AA	CC	CC	GG	AA	34.1	14.6
Wanmai29	GG	CC	CC	AA	GG	31.7	13.3
Wennong14	AA	CC	CC	AA	AA	32.7	14.0
Yannong15	AA	CC	CC	AA	AA	32.6	14.4
Yannong19	AA	CC	CC	AA	AA	33.7	14.3
Huapei5	AA	CC	CC	AA	GG	30.8	13.0
Yannong18	AA	CC	CC	AA	GG	31.1	13.9
Zhoumai13	AA	TT	CC	GG	AA	32.8	15.0
Zhoumai30	AA	CC	CC	GG	GG	31.2	13.1
Yumai57	GG	CC	CC	GG	GG	29.4	13.0
Zhoumai19	GG	CC	CC	AA	AA	32.0	14.6
Zhoumai31	AA	CC	CC	AA	GG	31.6	14.0
Zhengmai366	AA	CC	CC	AA	AA	30.8	15.5
Lumai14	GG	CC	CC	GG	AA	33.5	14.5
Jinan17	GG	CC	CC	GG	AA	35.0	12.9
Lumai9	GG	CC	CC	AA	GG	30.5	13.5
Xinong979-005	AA	CC	CC	AA	AA	33.5	14.7
Shan715	GG	CC	CC	AA	GG	31.3	13.6
Wunong148	GG	TT	CC	AA	GG	29.9	13.2
Yumai50	AA	CC	CC	AA	AA	32.1	14.2
Xinmai9408	GG	CC	CC	AA	GG	32.0	13.9
Bima4	GG	TT	CC	GG	GG	31.5	13.6
Linmai2	AA	TT	CC	GG	AA	36.3	16.0
Jishi02-1	AA	CC	CC	AA	AA	35.2	14.1
Shanmai509	AA	CC	CC	GG	AA	33.0	14.1
Shan512	AA	TT	CC	GG	AA	36.9	16.0
Sunong6	AA	CC	CC	GG	GG	35.6	16.1
Wanmai33	AA	TT	CC	GG	GG	35.7	15.9
Zhoumai28	AA	TT	CC	GG	GG	34.7	15.2
Gaoyou503	AA	CC	CC	AA	GG	34.6	15.1
Bima1	AA	TT	NA	GG	NA	33.2	15.1
Heng7228	AA	CC	NA	AA	NA	31.6	14.8
Huaimai21	AA	TT	NA	AA	NA	31.9	15.0
Jimai21	AA	TT	NA	AA	NA	33.5	15.1
Jinhe9123	AA	CC	NA	AA	NA	32.9	15.0
Zhoumai16	AA	CC	NA	GG	NA	31.6	14.0
Zhoumai22	AA	CC	NA	AA	NA	31.3	14.3
Fu936	AA	CC	NA	AA	NA	31.6	14.6
Gaocheng8901	AA	CC	NA	AA	NA	33.6	14.7
Jimai22	AA	CC	NA	AA	NA	32.3	14.4
Jinan13	AA	CC	NA	AA	NA	31.4	14.2
Liangxing66	AA	CC	NA	AA	NA	30.7	13.7
Liangxing99	AA	CC	NA	AA	NA	30.0	13.7
Linhan2	GG	CC	NA	AA	NA	28.6	13.6
Yumai63	AA	CC	NA	AA	NA	30.1	13.5
Zheng9023	AA	CC	NA	AA	NA	31.2	13.7
Zhengyin1	AA	CC	NA	AA	NA	30.1	13.2
Lumai5	AA	CC	NA	AA	NA	34.0	16.0
Neixiang5	AA	CC	NA	AA	NA	33.1	13.7
Zhengzhou3	AA	CC	NA	GG	NA	32.7	14.4
Shixin733	AA	CC	NA	AA	NA	31.1	13.2
Wanmai52	AA	CC	NA	AA	NA	32.5	13.5
Wanmai19	AG	CC	NA	AA	NA	31.9	14.3
Linmai4	GG	CC	NA	AA	NA	30.7	13.3
Shan229	AA	CC	NA	GG	NA	34.9	15.2
Taishan1	AA	TT	NA	GG	NA	35.3	15.2
Xinong88	AA	CC	NA	AA	NA	32.9	14.5
Yumai34	AA	TT	NA	AA	NA	34.4	15.1
Shan354	GG	TT	NA	AA	NA	34.6	14.6
Zhoumai18	GG	CC	NA	GG	NA	32.1	13.6
Shan253	GG	CC	NA	AA	NA	31.9	13.7
Shixin828	AA	CC	TT	AA	GG	31.4	13.6
Lumai11	AA	CC	TT	AA	GG	31.0	14.1
Luami15	GG	CC	TT	AA	GG	30.8	14.1
Lumai8	AA	CC	TT	AA	GG	31.3	14.3
Wanmai50	AA	TT	TT	GG	AA	33.5	15.0
Lankao906	AA	CC	TT	AA	AA	31.9	14.5
Lankao24	AA	CC	TT	AA	AA	32.4	14.4
Shannong78-59	AA	CC	TT	AA	GG	33.0	14.7
Zhongyu5	AG	TT	TT	AA	GG	30.8	13.6
Zhoumai11	AA	CC	TT	GG	GG	34.3	14.7
Lumai23	AA	CC	TT	AA	AA	32.6	13.9
Luohan2	GG	CC	TT	AA	AA	31.1	13.7
Shannong981	AA	TT	TT	AA	AA	31.0	13.4
Yanzhan4110	AA	CC	TT	GG	GG	29.3	15.2
Aikang58	GG	CC	TT	AA	GG	29.4	12.6
Luomai21	AA	CC	TT	AA	GG	31.9	13.8
Zhou8425B	AA	CC	TT	GG	GG	30.0	13.3
Bainong64	GG	CC	TT	GG	GG	30.9	13.1
Wan23094	AA	CC	TT	GG	GG	30.9	13.0
Yumai47	GG	CC	TT	AA	GG	30.9	13.7
Yumai49	GG	CC	TT	GG	GG	27.3	12.3
Jimai19	GG	CC	TT	GG	GG	30.4	12.7
Shanmai94	AA	CC	TT	AA	GG	30.3	13.3
Han6172	AA	CC	TT	AA	GG	27.8	11.8
Hengguan33	GG	CC	TT	AA	AA	32.6	14.9
Zhong892	AA	CC	TT	GG	AA	32.9	13.6
Jining16	GG	CC	TT	AA	AA	31.3	13.1
Zhoumai12	AA	CC	TT	GG	AA	33.6	14.3
Shanyou225	GG	CC	TT	GG	AA	34.6	13.4
Xinmai9	AA	CC	TT	AA	GG	33.0	14.3
Lumai7	AA	CC	TT	AA	GG	32.2	14.8

GPC, grain protein content; WGC, wet grain content.
